# Psychometric evaluation of the EQ-5D-Y-3L in Ethiopian pediatric inpatients: comparing self and proxy reports

**DOI:** 10.1186/s41687-025-00928-8

**Published:** 2025-08-13

**Authors:** Begashaw Melaku Gebresillassie, Yared Belete Belay, Adeladlew Kassie Netere, Ning Yan Gu

**Affiliations:** 1 School of Medicine and Public Health, University of Newcastle, Newcastle, Australia; 2 School of Pharmacy, University of Gondar, Gondar, Ethiopia; 3 School of Public Health and Preventive Medicine, Monash University, Melbourne, Australia; 4 Faculty of Pharmacy and Pharmaceutical Science, Monash University, Melbourne, Australia; 5 School of Nursing and Health Professions, University of San Francisco, California, USA

**Keywords:** EQ-5D-Y-3L, Responsiveness, Parent/caregiver-child/adolescent agreement, Ethiopia

## Abstract

**Background:**

Limited evidence exists regarding the measurement properties of the EQ-5D-Y-3L across different modes of administration. This study aimed to examine changes in parent/caregiver-child/adolescent dyad agreement concerning health-related quality of life (HRQoL) over time, assess variations in health status according to socio-demographic factors, and evaluate the responsiveness of the EQ-5D-Y-3L within a pediatric population in Ethiopia.

**Methods:**

The study was conducted at the University of Gondar Comprehensive Specialized Hospital, involving children/adolescents aged 4–18 years admitted to the pediatric inpatient unit. Children/adolescents completed the EQ-5D-Y-3L self-complete version at admission and discharge, while parents/caregivers completed the proxy version. Health status was analyzed utilizing the EQ-5D-Y-3L descriptive profiles, utility values, and the EuroQol Visual Analogue Scale (EQ VAS) scores, categorized by age, gender, and residence. Agreement between parent/caregiver and child/adolescent reports was evaluated using weighted Cohen’s kappa for dimension levels and the intraclass correlation coefficient (ICC) for utility and EQ VAS scores. Responsiveness was assessed through paired t-tests and the Paretian Classification of Health Change (PCHC) analysis, which classifies health status changes as improved, worsened, mixed, or unchanged based on changes across EQ-5D dimensions.

**Results:**

A total of 957 children/adolescents, with a mean age of 10.7 ± 4.3 years, along with their parents/caregivers, participated in the study. The predominant diagnoses included pneumonia, meningitis, malaria, malnutrition, and glomerulonephritis. Both child/adolescent and parent/caregiver reports indicated poorer health status among older adolescents (13–18 years), boys from rural areas. Agreement on the EQ-5D-Y-3L dimension levels was fair to moderate at admission (weighted kappa ranging from 0.28 to 0.38) and was poorer at discharge for the ‘worried, sad or unhappy’ dimension (weighted kappa of 0.15). Agreement on utility and EQ VAS scores was acceptable at both admission and discharge (ICC: 0.498–0.676), with moderate to good agreement observed among children/adolescents aged 7–16 years. However, agreement on utility scores decreased at discharge for older boys (13–18 years old) and urban residents, while it increased for the younger age group (4–6 years old). Responsiveness analysis demonstrated significant improvements in the dimensions of ‘Looking After Myself,’ ‘Mobility,’ and ‘Worried, Sad or Unhappy,’ with most children/adolescents exhibiting health improvements according to PCHC criteria.

**Conclusion:**

Parent/caregiver-child/adolescent dyad agreement concerning HRQoL was low to moderate and varied according to socio-demographic factors. The EQ-5D-Y-3L instrument demonstrated responsiveness to changes in health status, supporting its utility in pediatric populations. These findings underscore the importance of employing age-appropriate and context-sensitive HRQoL assessment tools in pediatric care and health policy. Incorporating both child/adolescent and parent/caregiver perspectives can inform clinical decisions and resource allocation, especially in low-resource settings. Further research is warranted to explore factors influencing these variations and to enhance understanding of their underlying causes.

## Background

The advancement of children’s health is a paramount concern for the World Health Organization (WHO) and its member nations. Accurate and effective methods to assess children’s health status are essential for evaluating the impact of health interventions on the health-related quality of life (HRQoL) of sick children. Over recent decades, new and emerging HRQoL measures have been developed to assess children’s HRQoL, focusing on their physical, mental, social, and psychological well-being [1–6]. These measures aim to reflect subjective perceptions of health across various dimensions and are measured using various modes of administration.

The EQ-5D Youth Version with 3 Levels (EQ-5D-Y-3L) is one of the instruments developed by the EuroQol Research Foundation to assess HRQoL in pediatric populations [7]. Adapted from the adult EQ-5D-3L, the EQ-5D-Y-3L assesses five health domains: mobility (MO), looking after myself (LAM), usual activity (UA), pain/discomfort (PD), and worried, sad or unhappy (WSU), using a 3-point Likert scale: (1) no problems, (2) some problems, and (3) a lot of problems. It also includes a EuroQol Visual Analogue Scale (EQ VAS), ranging from 0 (worst imaginable health) to 100 (best imaginable health), for overall health assessment [7].

According to the EuroQol EQ-5D-Y-3L user guide [8], this instrument should be used for children/adolescents aged 8 to 15 years. For children aged 4–7, a proxy version should be used; for those aged 12 to 15 years, both the youth and adult EQ-5D versions can be used, although the youth version is preferred. For children 16 years and older, the adult version of the EQ-5D instruments is recommended. The user guide also emphasizes the need to verify when children can effectively and reliably report their health status [8].

Direct self-report in HRQoL assessment minimizes the potential biases and misinterpretations that may arise from proxy responses, including children [9]. In real-world practices, assessing young children’s HRQoL often relies on proxy reports from parents or caregivers [10–12]. This is mainly due to the presumed cognitive and linguistic limitations in children [10–12]. However, emerging evidence suggests that children younger than 8 years can competently engage with HRQoL measures [13, 14]. Discrepancies between child self-reports and proxy reports highlight the importance of capturing and understating both perspectives. There are studies that indicated varying levels of agreement across different HRQoL domains between child and proxy reports [8, 9, 15]. Furthermore, factors like family structure, socioeconomic status, and parental well-being significantly influence proxy-reported HRQoL [16–19]. Studies comparing the agreement between self-reports and proxy-reports have shown mixed results on the construct validity of the EQ-5D-Y-3L [9, 20, 21]. These findings underscore the need for further investigation into the measurement properties of responses obtained from different administration methods (e.g., proxy vs. self-report).

Despite its growing use, evidence on the psychometric properties of the EQ-5D-Y-3L, particularly validation efforts in low-income settings, remains limited [22–24]. While studies have been conducted in various high-income countries, there is a notable scarcity of research examining its performance in diverse cultural and socioeconomic contexts prevalent in low-income regions [25]. This gap is significant because the generalizability of HRQoL measures can be impacted by cultural interpretations of health, differing healthcare access realities, and socio-economic determinants of health. For instance, concepts of well-being and illness expression can vary across cultures, potentially influencing how children and proxies report health states [26]. Furthermore, healthcare access disparities and the common burden of communicable diseases in low-income settings like Ethiopia may lead to unique health profiles and different perceptions of health problems compared to high-income settings [27, 28]. These factors can affect the agreement between self and proxy reports, as caregivers’ experiences with limited healthcare resources may shape their perspectives on their child’s health status differently from the child’s own experience [27, 28].

This study aimed to compare the self-report by children/adolescents and the proxy responses by the caregivers/parents in Ethiopia. Notably, this age range is wider than the age range recommended by the EuroQol EQ-5D-Y-3L user guide for each administration method. The goal was to gain a better understanding of the age appropriateness of the implementation of the EQ-5D-Y-3L in Ethiopian children/adolescents. Specifically, the study sought to answer the following questions: (1) What is the level of agreement between child/adolescent self-reports and parent/caregiver proxy reports of HRQoL? (2) How does reported HRQoL vary across socio-demographic characteristics such as age, gender, and residence? (3) Is the EQ-5D-Y-3L responsive to changes in health status during hospitalisation?

## Methods

### Study design and setting

From June 01 to October 31, 2023, repeated measurements were collected at the University of Gondar Comprehensive Specialized Hospital (UOGCSH) in Ethiopia. The modified Amharic version of the EQ-5D-Y-3L, along with the EQ VAS, was administered to parents/caregivers and children/adolescents aged 4 to 18 years, first at admission (Time 1) and then at patient discharge (Time 2). This hospital is a specialized referral hospital in the region and receives referrals from an area that encompasses 17 million people, of which 43% are aged < 14 years, and 88% live in rural areas [29, 30].

### Study population and sample size

Sick children/adolescents 4–18 years of age, diagnosed with prevalent acute illnesses and admitted into the University Hospital in Ethiopia, were eligible. These prevalent and acute illnesses in the region included, for instance, respiratory infections, diarrhea, meningitis, malaria, and severe acute malnutrition. These health conditions were selected based on their significant impact on pediatric morbidity and mortality in the region, as documented in prior research, and they reflected the high prevalence and impact on HRQoL in this demographic [30]. In addition to the children/adolescents, their parents/caregivers were also included for proxy responses. Children/adolescents who had severe conditions such as a low level of consciousness, disorientation, or impairment in vision were excluded from the study to ensure data quality on self-reported responses.

The sample size was calculated using a two-sample t-test to detect a small, meaningful difference (µ = 0.05) in the mean EQ-5D-Y-3L index score across the two administration methods (child/adolescent vs. parent/caregiver reports) within each age group (4–18 years) [31, 32], with 80% power and a significance level of 0.05. Based on these assumptions, the computed sample size per age category was 64, resulting in a minimum total required sample size of *n* = 960. By adding 25 participants to account for potential non-response, the final sample size was determined to be 985 [33]. The child/adolescent-parent/caregiver pairs were recruited using a quota sampling strategy, with participants enrolled consecutively within each age category until the target sample size for that age category was achieved.

### Data collection

Data was collected from June 01 to October 31, 2023, by qualified nurses who had received specific training on the study procedures. For baseline data collected at admission, parents/caregivers completed detailed questionnaires that captured socio-demographic characteristics, including patients’ age, gender, educational background, household residence, and income. They also completed the Amharic proxy version of the EQ-5D-Y-3L, along with EQ VAS translated by the EuroQol Group.

Meanwhile, children or adolescents aged 8–18 years filled out the self-complete Amharic versions of the EQ-5D-Y-3L and EQ VAS, translated by the EuroQol group. For younger children (aged 4–7 years), the questionnaires were also self-completed with assistance: trained data collectors read the instructions and each question aloud exactly as written, provided simple clarifications, when necessary, without altering item meanings, and ensured that the children understood the content before responding. In all cases, the responses recorded were those of the children themselves.

Follow-up data were collected just before the patients’ discharge post-treatment, following the same method as baseline and documenting the changes in their HRQoL. Furthermore, clinical diagnosis and length of hospital stay were collected from patients’ medical records. These records were carefully reviewed and verified by a senior pediatrician to ensure accuracy. The interviews with children/adolescents were conducted at their bedside in the pediatric wards. Parent/caregiver interviews, on the other hand, took place in a designated nurses’ room to ensure comfort and privacy.

### Analyses

The data was entered into Excel and subsequently imported into STATA version 17 for cleaning and analysis. The normality assumption was checked prior to analysis. Descriptive statistics were used to summarize the demographic and clinical characteristics of the study participants. Data were presented as frequencies, percentages, means with standard deviations, or medians with interquartile ranges, depending on the distribution of the data. In this analysis, EQ-5D-Y-3L dimension level 1 (“no problems”) was classified as ‘no problems,’ while levels 2 (“some problems”) and 3 (“a lot of problems”) were classified as ‘problems.’ The chi-square test was used to compare the proportions of reported ‘problems,’ with a threshold of alpha = 0.05 considered indicative of statistical significance.

Agreement between child/adolescent self-reports and parent/caregiver proxy reports was evaluated using percent agreement and weighted Cohen’s kappa (κ) for categorical dimensions. Intraclass correlation coefficients (ICCs) were used to assess agreement on continuous outcomes (EQ VAS and index scores). Convergent validity was assessed using Spearman’s rank correlation coefficient between EQ VAS and index scores. Interpretation thresholds were as follows: For Cohen’s kappa: ≤ 0 = poor, 0.01–0.20 = slight, 0.21–0.40 = fair, 0.41–0.60 = moderate, 0.61–0.80 = substantial, and > 0.81-1 = almost perfect agreement [34]; for ICC: < 0.5 = poor, 0.5–0.75 = moderate, 0.75–0.90 = good, and > 0.90 = excellent agreement [35]; and for Spearman correlation: < 0.4 = weak, 0.4–0.69 = moderate, and ≥ 0.7 = strong correlation [36].

Responsiveness in detecting changes in health status over time was examined at both the EQ-5D-Y-3L dimension level, analyzed using percent reduction of problems, and at the individual level, using the Paretian Classification of Health Change (PCHC) analysis. According to PCHC analysis, an EQ-5D-Y-3L health state is considered ‘better’ than another if it is better on at least one dimension and no worse on any other dimension [37]. The paired t-test was applied to assess further responsiveness, comparing mean EQ-5D-Y-3L index and EQ VAS scores between baseline at admission and follow-up at discharge. Due to the unavailability of a specific EQ-5D-3L value set for Ethiopia, we applied the Zimbabwe value set to compute the EQ-5D-Y-3L index score [38]. This decision was based on geographic proximity, socio-economic similarities, and shared regional health challenges between Zimbabwe and Ethiopia.

In addition, we used the treating physicians’ documentation of clinical progress prior to discharge as a benchmark to anchor the responsiveness analysis. These physician-recorded improvements, based on routine clinical assessments, were consistently observed for all participants included in the analysis. This approach facilitated an objective evaluation of the EQ-5D-Y-3L’s sensitivity to change over time, allowing us to assess the alignment between reported HRQoL improvements and clinical recovery.

## Results

A total of 985 pediatric inpatients and their parents/caregivers participated in both the baseline and follow-up interviews. However, 28 dyad responses were excluded due to incomplete data on the descriptive system or the EQ VAS in one of the EQ-5D-Y-3L versions, either at baseline or follow-up. As a result, the analysis included data from 957 (97.1%) dyads.

**Fig. 1:**
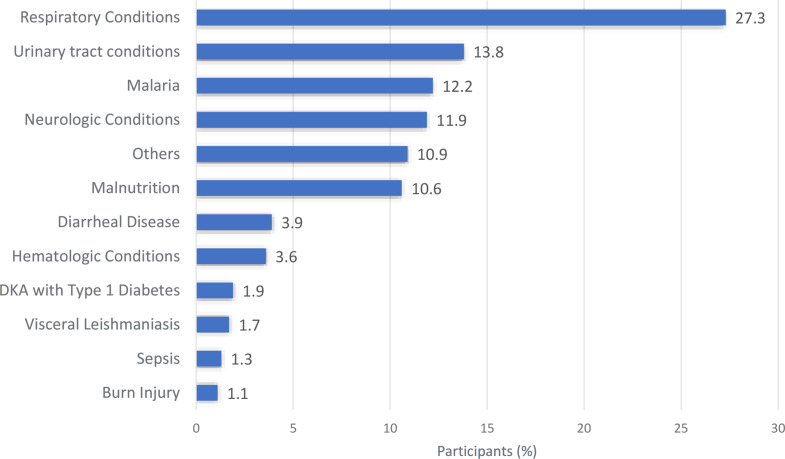
Distribution of admission diagnoses among children and adolescents (*N* = 957). DKA: Diabetic ketoacidosis

The mean age of the children/adolescents was 10.7 years (SD: 4.3, range: 4–18 years). Of the total participants, 52.7% were boys (*n* = 504) and 47.3% were girls (*n* = 453). Most participants resided in rural areas (81.3%, *n* = 778), and over half of the children were from households reporting insufficient income (69.1%, *n* = 661). The most prevalent health conditions included respiratory tract infections including pneumonia and pharyngitis (27.3%, *n* = 261), genitourinary conditions such as urinary tract infections (UTIs) and glomerulonephritis (13.8%, *n* = 132), and malaria infection (12.2%, *n* = 117). A statistically significant difference was observed in parent/caregiver education levels between urban and rural settings: 50.5% of urban parents/caregivers had completed at least primary school, compared to only 17.7% of rural parents/caregivers (*p* < 0.001) (Table 1; Fig. 1).

**Table 1:** Demographic and clinical characteristics of the study participants

Characteristics		Children/adolescents
Age (years), mean (SD)		10.7(4.3)
Gender, n(%)	Male	504(52.7)
	Female	453(47.3)
Education status, n(%)	Pre-primary	212 (22.2)
	Primary (Grade 1–8)	723 (75.5)
	Secondary (Grade 9–10)	22 (2.3)
Residence, n(%)	Urban	179(18.7)
	Rural	778(81.3)
Nutritional status, n(%)	Underweight	290(30.3)
	Normal	662(69.2)
	Overweight/obese	5(0.5)
Top diagnosis, n(%)	Respiratory conditions	261(27.3)
	Genitourinary conditions	132(13.8)
	Malaria	117(12.2))
	Neurologic conditions	114(11.9)
	Malaria	101(10.6)
Length of hospital stay in days, median (IQR)		10(7–15)
		**Caregiver/proxy**
Age (years), mean (SD)		40.4(8.7)
Gender, n(%)	Male	558(58.3)
	Female	399(41.7)
Education status, n(%)	No formal education	721(75.3)
	Primary (Grade 1–8)	115(12.0)
	Secondary (Grade 9–10)	57(6.0)
	College and above	64(6.7)
Household income, n(%)	Enough	296(30.9)
	Not enough	661(69.1)

Household income – which is self-reported by participants

The child/adolescent-reported data highlighted an increase in HRQoL problems with advancing age, particularly noted in the dimensions of LAM (*p* = 0.019), UA (*p* < 0.001), PD (*p* < 0.001) and WSU (*p* < 0.001), with the oldest age group [12–18] consistently reporting the highest frequency of problems (34.4% in LAM, 33.5% in UA, 35.2% in PD, and 26.1% in WSU). Similarly, parent/caregiver-reported problems also demonstrated an increasing trend by the age group, though these differences did not reach statistical significance. Gender differences were not statistically significant in most dimensions; however, both children/adolescents (*p* = 0.009) and parents/caregivers (*p* < 0.001) reported a significant impact of household income on the emotional well-being of children. Specifically, children/adolescents from families with inadequate income were reported to have more problems in the WSU dimension. Moreover, urban versus rural residence showed a statistically significant difference in the mobility dimension of child/adolescent reported measures, suggesting environmental factors may influence physical health components (Table 2).

**Table 2:** Number (percentages) of children/adolescents and parents/caregivers with problems at admission by socio-demographic characteristics

		Child/Adolescent age			*P*-value	Child/adolescent gender		*P*-value	Residence		*P*-value	Child/adolescent nutrition status		*P*-value	Household income		*P*-value
**Child/Adolescent Report**																	
	**%**	**4**–**7**	**8**–**12**	**12**–**18**		**Boys**	**Girls**		**Urban**	**Rural**		**Under weight**	**Normal/over-** **weight**		**Enough**	**Not Enough**	
MO	No problems	35 (3.6)	32 (3.3)	39 (4.1)	*0.517*	48 (5.0)	58 (6.1)	*0.106*	28 (2.9)	78 (8.1)	0.031*	29 (3.0)	77 (8.0)	*0.484*	30 (3.1)	76 (7.9)	*0.535*
	Problems	238 (24.9)	291 (30.4)	322 (33.6)		456 (47.6)	395 (41.3)		151 (15.8)	700 (73.1)		261 (27.3)	590 (61.6)		266 (27.8)	585 (61.1)	
LAM	No problems	44 (4.6)	44 (4.6)	32 (3.3)	0.019*	59 (6.2)	61 (6.4)	*0.412*	28 (2.9)	92 (9.6)	*0.164*	28 (2.9)	92 (9.6)	*0.076*	39 (4.1)	81 (8.5)	*0.691*
	Problems	229 (23.9)	279 (29.1)	329 (34.4)		445 (46.5)	392 (41.0)		151 (15.8)	686 (71.7)		262 (27.4)	575 (60.1)		257 (26.8)	580 (60.6)	
UA	No problems	62 (6.5)	39 (4.1)	40 (4.2)	< 0.001*	80 (8.3)	61 (6.4)	*0.294*	29 (3.0)	112 (11.7)	*0.539*	40 (4.2)	101 (10.5)	*0.588*	52 (5.4)	89 (9.3)	*0.098*
	Problems	211 (22.0)	284 (29.7)	321 (33.5)		424 (44.3)	392 (41.9)		150 (15.7)	666 (69.6)		250 (26.1)	566 (59.1)		244 (25.5)	572 (59.8)	
PD	No problems	69 (7.2)	38 (4.0)	24 (2.5)	< 0.001*	72 (7.5)	59 (6.2)	*0.571*	23 (2.4)	108 (11.3)	*0.717*	45 (4.7)	86 (9.0)	*0.278*	41 (4.3)	90 (9.4)	*0.922*
	Problems	204 (21.3)	285 (28.8)	337 (35.2)		432 (45.1)	394 (41.2)		156 (16.3)	670 (70.0)		245 (25.6)	581 (60.7)		255 (26.6)	571 (59.7)	
WSU	No problems	127 (13.3)	138 (14.4)	111 (11.6)	< 0.001*	211 (22.0)	165 (17.2)	*0.085*	75 (7.8)	301 (31.4)	*0.428*	113 (11.8)	263 (27.5)	*0.892*	98 (10.2)	278 (29.0)	0.009*
	Problems	146 (15.2)	185 (19.3)	250 (26.1)		293 (30.6)	288 (30.1)		104 (10.8)	477 (49.8)		177 (18.5)	404 (42.2)		198 (20.7)	383 (40.0)	
EQ VAS	Mean (SD)	36.1 (20.5)	36.8 (17.2)	35.7 (17.5)	*0.701*	36.0 (18.5)	36.4 (18.1)	*0.756*	38.0 (19.2)	35.7 (18.0)	*0.134*	34.9 (18.5)	36.7 (18.2)	*0.143*	32.7 (19.5)	37.7 (17.5)	< 0.001*
**Caregiver/Proxy Report**																	
MO	No problems	30 (3.1)	47 (4.9)	36 (3.8)	*0.159*	67 (7.0)	46 (4.8)	*0.133*	25 (2.6)	88 (9.2)	*0.321*	35 (3.6)	78 (8.1)	*0.869*	31 (3.2)	82 (8.6)	*0.392*
	Problems	243 (25.4)	276 (28.8)	325 (34.0)		437 (45.7)	407 (42.5)		154 (16.1)	690 (72.1)		255 (26.6)	589 (61.5)		265 (27.7)	579 (60.5)	
LAM	No problems	30 (3.1)	52 (5.4)	52 (5.4)	*0.193*	76 (7.9)	58 (6.1)	*0.311*	24 (2.5)	110 (11.5)	*0.799*	44 (4.6)	90 (9.4)	*0.491*	44 (4.6)	90 (9.4)	*0.607*
	Problems	243 (25.4)	271 (28.3)	309 (32.3)		428 (44.7)	395 (41.3)		155 (16.2)	668 (69.8)		246 (25.7)	577 (60.3)		252 (26.3)	571 (59.7)	
UA	No problems	35 (3.6)	52 (5.4)	64 (6.7)	*0.240*	81 (8.5)	70 (7.3)	*0.793*	21 (2.2)	130 (13.6)	*0.100*	52 (5.4)	99 (10.3)	*0.228*	46 (4.8)	105 (11.0)	*0.893*
	Problems	238 (24.9)	271 (28.3)	297 (31.0)		423 (44.2)	383 (40.0)		158 (16.5)	648 (67.7)		238 (24.9)	568 (59.3)		250 (26.1)	556 (58.1)	
PD	No problems	42 (4.4)	43 (4.5)	44 (4.6)	*0.503*	76 (7.9)	53 (5.5)	*0.126*	17 (1.8)	112 (11.7)	*0.084*	44 (4.6)	85 (8.9)	*0.312*	36 (3.8)	93 (9.7)	*0.425*
	Problems	231 (24.1)	280 (29.2)	317 (33.1)		428 (44.7)	400 (41.8)		162 (16.9)	666 (69.6)		246 (25.7)	582 (60.8)		260 (27.2)	568 (59.3)	
WSU	No problems	96 (10.0)	120 (12.5)	117 (12.2)	*0.425*	193 (20.2)	140 (14.6)	0.017*	72 (7.5)	261 (27.3)	*0.091*	102 (10.6)	231 (24.1)	*0.872*	79 (8.2)	254 (26.5)	< 0.001*
	Problems	177 (18.5)	203 (21.2)	244 (25.5)		311 (32.5)	313 (32.7)		107 (11.2)	517 (54.0)		188 (19.6)	436 (45.5)		217 (22.7)	407 (42.5)	
EQ VAS	Mean (SD)	33.5 (19.3)	36.5 (16.0)	38.1 (18.9)	0.009*	35.4(18.4)	37.3(17.9)	*0.104*	37.5(18.4)	36.0(18.1)	*0.294*	34.7(18.8)	37.0(17.8)	*0.073*	33.0(21.0)	37.7(16.6)	< 0.001*

Note: MO: Mobility; LAM: Looking After Myself; UA: Doing Usual Activities; P/D: Pain/Discomfort; WSU: Feeling Worried, Sad or Unhappy; SD: Standard deviation; NB: Nutritional status was identified by physician assessment; Household income– which is self-reported by parents/caregivers; Percentages are calculated based on the total number of participants (*N* = 957); P-values were calculated using Chi-square tests for EQ-5D-Y-3L dimensions and Welch’s t-tests for EQ VAS scores

### Agreement between child/adolescent and caregiver measures

At both baseline and follow-up, the level of agreement on EQ-5D dimension levels was fair (weighted kappa 0.28 to 0.38), except at the discharge for the WSU dimension, which showed poor agreement (weighted kappa 0.15). In all EQ-5D-Y-3L dimensions, the percentage of exact agreement increased from baseline to follow-up, indicating improved alignment between child/adolescent and parent/caregiver reports after hospitalization. However, weighted kappa values were generally higher at baseline, with kappa coefficients ranging from 0.28 to 0.38, except for the UA dimension (Table 3). The overall agreement level on EQ-5D-Y-3L utility values and EQ VAS scores at both admission and discharge was in the acceptable range, with EQ-5D-Y-3L index ICCs of 0.582 (95% CI: 0.518–0.638) and 0.498 (95% CI: 0.421–0.564), respectively. Similarly, EQ VAS scores exhibited agreement with ICC values of 0.671 (95% CI: 0.627–0.711) at admission and 0.676 (95% CI: 0.570–0.750) at discharge (Table 4).

Gender-based analysis revealed varying agreement levels. Boys exhibited ICC values of 0.625 (95% CI: 0.550–0.687) and 0.493 (95% CI: 0.388–0.579) for EQ-5D-Y-3L index at admission and discharge, respectively. The corresponding EQ VAS scores displayed ICC values of 0.689 (95% CI: 0.630–0.739) at admission and 0.688 (95% CI: 0.557–0.771) at discharge. Girls, on the other hand, demonstrated slightly lower ICC values for both EQ-5D-Y-3L index and EQ VAS scores, with index ICCs of 0.537 (95% CI: 0.436–0.618) at admission and 0.501 (95% CI: 0.397–0.586) at discharge. Their EQ VAS score agreement was comparable, with ICCs of 0.650 (95% CI: 0.579–0.709) at admission and 0.659 (95% CI: 0.553–0.735) at discharge. Similarly, child/adolescent age-stratified analysis demonstrated varying agreement patterns. Younger children (ages 4–6) and older adolescents (ages 16–18) displayed lower ICC values, indicating lower agreement, while children/adolescents aged 7 to 15 years exhibited higher ICC values, suggesting more consistent agreement between child/adolescent and parent/caregiver reports. Residence-based comparisons between urban and rural settings revealed rural residents consistently displayed slightly higher ICCs compared to their urban counterparts, particularly for the EQ-5D-Y-3L index and EQ VAS scores at discharge, indicating more consistent health status reporting in rural settings (Table 4).

**Table 3:** Agreement between child/adolescent and parent/caregiver reported health status by EQ-5D-Y-3L dimensions

EQ-5D-Y-3L Dimensions	Time point	Percent exact agreement	Weighted kappa coefficient
MO	Baseline	78.9	0.38
	Follow up	88.4	0.34
LAM	Baseline	79.0	0.38
	Follow up	88.3	0.29
UA	Baseline	74.4	0.28
	Follow up	86.9	0.31
PD	Baseline	74.0	0.28
	Follow up	84.5	0.26
WSU	Baseline	72.8	0.36
	Follow up	89.5	0.15

**Table 4:** Agreement between child/adolescent and caregiver reported health status by EQ-5D-Y-3L index and EQ VAS scores

HRQoL	Frequency (%)	EQ-5D-Y-3L-index ICC (95% CI)		EQ VAS score ICC (95% CI)	
		Admission	Discharge	Admission	Discharge
Total	957(100)	0.582 (0.518–0.638)	0.498 (0.421–0.564)	0.671 (0.627–0.711)	0.676 (0.570–0.750)
**Gender**					
Boys	504 (52.7)	0.625 (0.550–0.687)	0.493 (0.388–0.579)	0.689 (0.630–0.739)	0.688 (0.557–0.771)
Girls	453 (47.3)	0.537 (0.436–0.618)	0.501 (0.397–0.586)	0.650 (0.579–0.709)	0.659 (0.553–0.735)
Child/adolescent 4 age	75 (7.8)	0.183 (0.149–0.440)	0.401 (0.054–0.622)	0.642 (0.436–0.773)	0.130 (-0.164-0.379)
5	69 (7.2)	0.139 (0.262–0.431)	0.378 (0.027–0.607)	0.489 (0.180–0.682)	0.161 (-0.164-0.425)
6	60 (6.3)	0.351 (-0.036-0.601)	0.511 (0.153–0.715)	0.554 (0.253–0.734)	0.525 (0.094-739)
7	69 (7.2)	0.787 (0.656–0.868)	0.588 (0.338–0.744)	0.667 (0.461–0.794)	0.646 (0.421–0.783)
8	64 (6.7)	0.509 (0.196–0.712)	0.282 (-0.157-0.558)	0.619 (0.375–0.768)	0.745 (0.571–0.848)
9	69 (7.2)	0.831 (0.728–0.895)	0.316 (-0.084-0.571)	0.837 (0.737–0.899)	0.820 (0.705–0.889)
10	64 (6.7)	0.796 (0.663–0.876)	0.706 (0.503–0.824)	0.792 (0.656–0.873)	0.866 (0.781–0.919)
11	61 (6.4)	0.725 (0.544–0.834)	0.591 (0.321–0.754)	0.462 (0.107–0.677)	0.893 (0.822–0.935)
12	65 (6.8)	0.739 (0.571–0.841)	0.764 (0.594–0.860)	0.792 (0.659–0.873)	0.890 (0.809–0.936)
13	63 (6.6)	0.700 (0.504–0.819)	0.622 (0.376–0.771)	0.775 (0.629–0.864)	0.831 (0.721–0.898)
14	58 (6.1)	0.793 (0.651–0.877)	0.603 (0.332–0.764)	0.744 (0.567–0.848)	0.694 (0.480–0.820)
15	75 (7.8)	0.590 (0.350–0.741)	0.440 (0.110–0.647)	0.855 (0.769–0.909)	0.910 (0.857–0.944)
16	52 (5.4)	0.590 (0.281–0.765)	0.491 (0.108–0.709)	0.655 (0.400-0.802)	0.786 (0.624–0.877)
17	58 (6.1)	0.445 (0.088–0.666)	0.185 (-0.389-0.520)	0.597 (0.319–0.762)	0.522 (0.191–0.717)
18	55 (5.7)	0.607 (0.323–0.772)	0.050 (-0.547-0.429)	0.474 (0.104–0.692)	0.253 (-0.249-0.558)
Residence Urban	179 (18.7)	0.574 (0.417–0.687)	0.457 (0.264–0.599)	0.683 (0.575–0.764)	0.552 (0.229–0.720)
Rural	778 (81.3)	0.585 (0.517–0.642)	0.503 (0.422–0.572)	0.668 (0.617–0.711)	0.705 (0.626–0.764)

95%CI: 95% confidence interval; HRQoL: Health related quality of life; ICC: Intraclass correlation coefficient

### Convergent validity and responsiveness

The convergent validity analysis showed that correlations between the EQ-5D-Y-3L index and EQ VAS scores were statistically significant (*p* < 0.01) for both child/adolescent and parent/caregiver reports, indicating a consistent and moderate positive relationship within each group. Specifically, the correlation coefficient for child/adolescent reports was 0.453 at admission and increased to 0.514 at discharge/follow-up. Parent/caregiver reports mirrored these findings, with correlation coefficients of 0.512 at admission and 0.520 at discharge, underscoring the parent’s/caregiver’s consistent perception of the child’s/adolescent’s health.

**Fig. 2:**
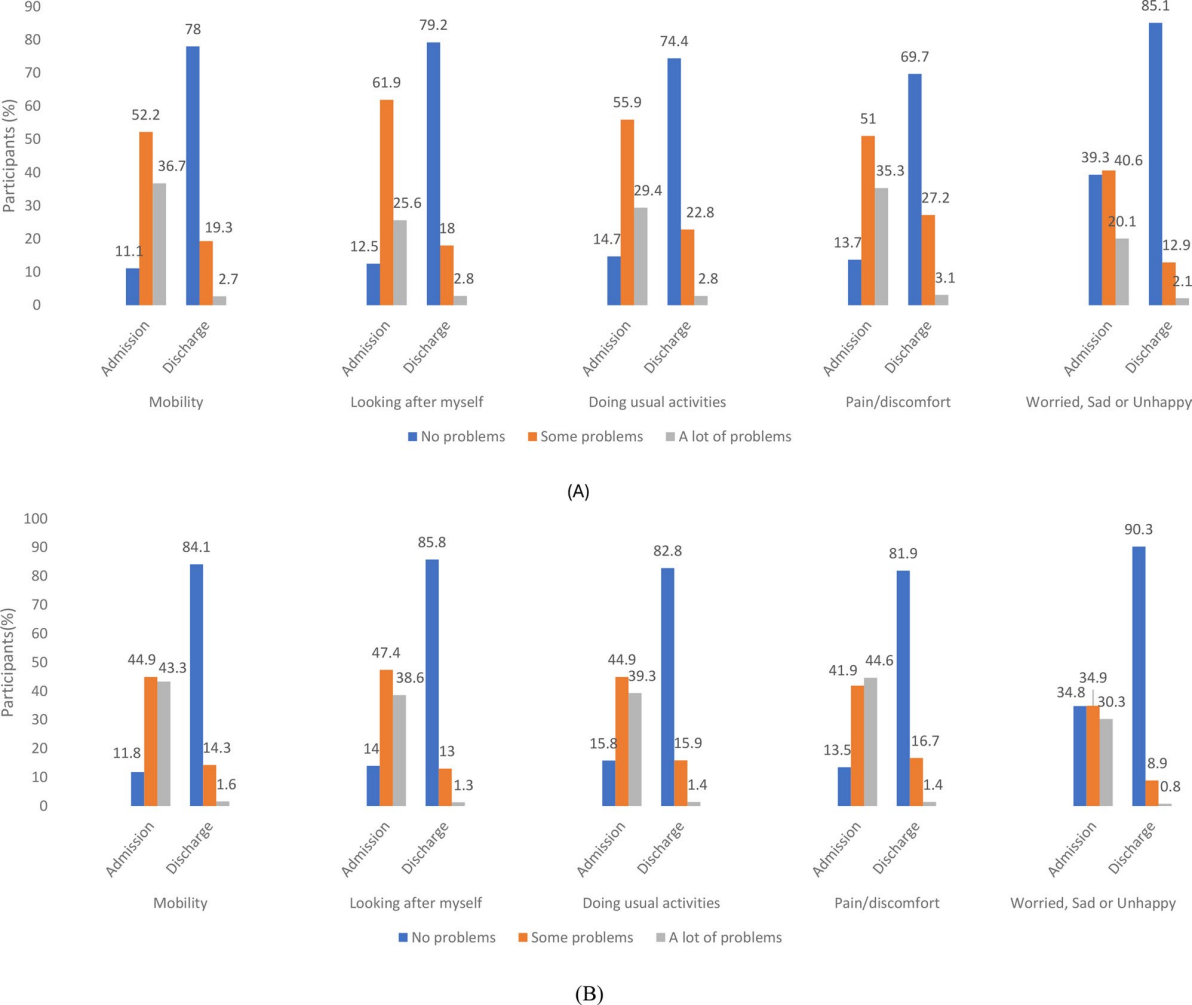
Distribution of health problems across EQ-5D-Y-3L dimensions at admission and discharge, comparing (**A**) child/adolescent self-reports and (**B**) parent/caregiver reports. Bars represent the percentage of participants selecting each level: “No problems,” “Some problems,” and “A lot of problems”

**Fig. 3:**
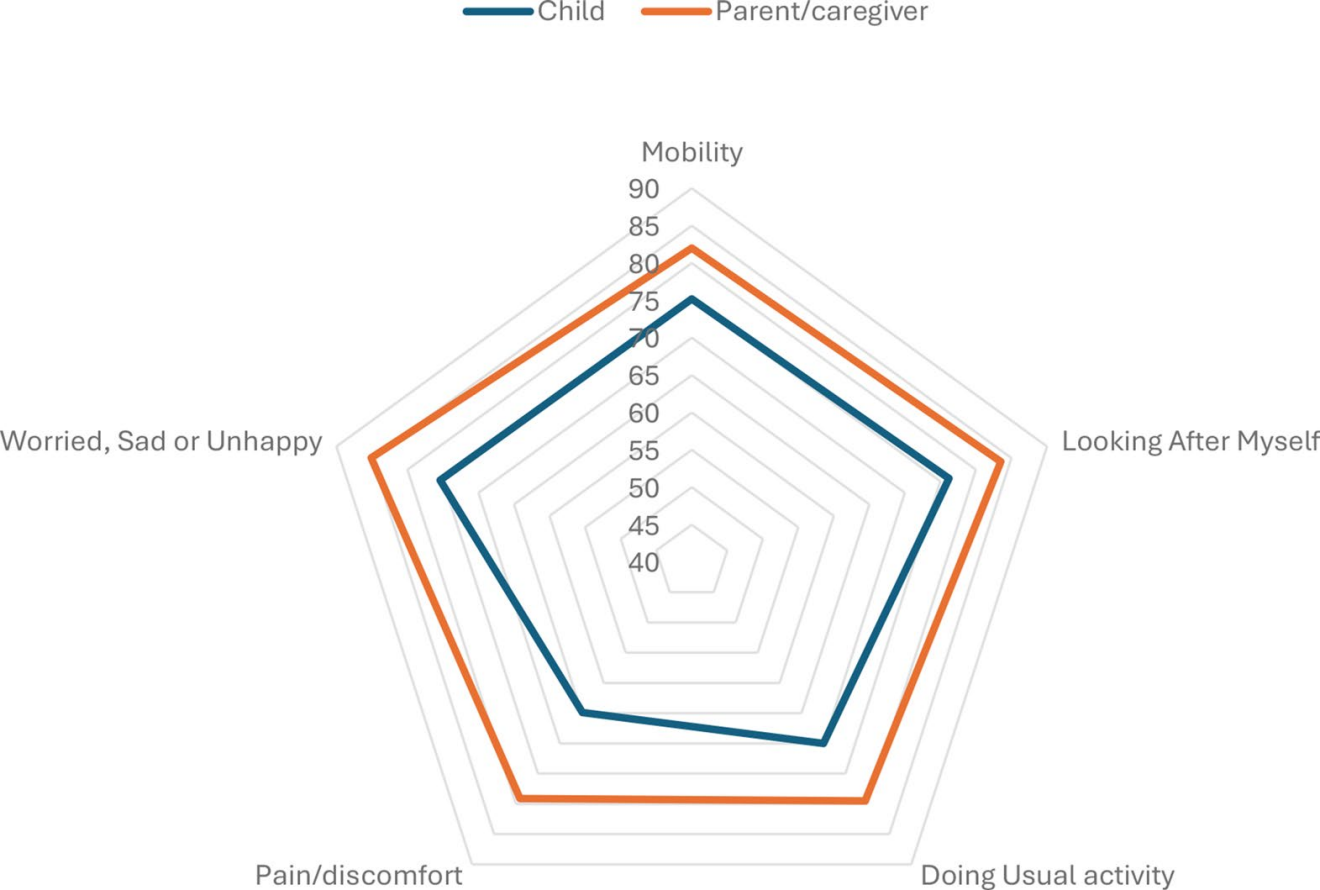
Change in the percentage of reported problems between admission and discharge by respondent type (child/adolescent vs. parent/caregiver)

Our analysis, which examined the responsiveness of both child/adolescent and parent/caregiver reported measures against the documented health improvements, yielded consistent results. Both children/adolescents and parents/caregivers reported substantial improvements across all EQ-5D-Y-3L dimensions from admission to discharge (Fig. 2). Among child/adolescent self-reports, the greatest reduction in reported problems was observed in the LAM dimension, with a 76.2% decrease, followed closely by MO and WSU, which showed reductions of 75.2% and 75.4%, respectively. In contrast, parent/caregiver reports indicated even greater improvements. The WSU dimension showed the most pronounced change, with an 85.1% reduction, followed by LAM (83.5%) and MO (82.0%) (Tables 5 and 6; Fig. 3).

**Table 5:** Child/adolescent reported problems by EQ-5D-Y dimensions at admission and discharge

Level	EQ-5D-Y-3L dimensions									
	Mobility (%)		Looking After Myself (%)		Doing Usual activity (%)		Pain/discomfort (%)		Worried, Sad or Unhappy (%)	
	Admission	Discharge	Admission	Discharge	Admission	Discharge	Admission	Discharge	Admission	Discharge
**1**	106 (11.1)	746 (78.0)	120 (12.5)	758 (79.2)	141 (14.7)	712 (74.4)	131 (13.7)	667 (69.7)	376 (39.3)	814 (85.1)
**2**	500 (52.2)	185 (19.3)	592 (61.9)	172 (18.0)	535 (55.9)	218 (22.8)	488 (51.9)	260 (27.2)	389 (40.6)	123 (12.8)
**3**	351 (36.7)	26 (2.7)	245 (25.6)	27 (2.8)	281 (29.4)	27 (2.8)	338 (35.3)	30 (3.1)	192 (20.1)	20 (2.1)
Total	957 (100)	957 (100)	957 (100)	957 (100)	957 (100)	957 (100)	957 (100)	957 (100)	957 (100)	957 (100)
Number reporting presence of problems	851 (88.9)	211 (22.0)	837 (87.5)	199 (20.8)	816 (85.3)	245 (25.6)	826 (86.3)	290 (30.3)	581 (60.7)	143 (14.9)
Change in numbers reporting presence of problems (%)	-640 (-75.2)		-638 (-76.2)		-571 (-70.0)		-536 (64.9)		-438 (-75.4)	
Rank of dimensions in terms of % changes	3		1		4		5		2	

Level 1 = no problems, Level 2 = some problems, and Level 3 = a lot of problems. Percentages are calculated based on the total number of participants (*N* = 957) at each time point. Negative percentages in the “Change in numbers reporting presence of problems (%)” indicate reductions in reported problems from admission to discharge

**Table 6:** Parent/caregiver reported problems by EQ-5D-Y dimensions at admission and discharge

Level	EQ-5D-Y-3L dimensions									
	Mobility (%)		Looking After Myself (%)		Doing Usual Activity (%)		Pain/Discomfort (%)		Worried, Sad or Unhappy (%)	
	Admission	Discharge	Admission	Discharge	Admission	Discharge	Admission	Discharge	Admission	Discharge
**1**	113 (11.8)	805 (84.1)	134 (14.0)	821 (85.8)	151 (15.8)	792 (82.8)	129 (13.5)	784 (81.9)	333 (34.8)	864 (90.3)
**2**	430 (44.9)	137 (14.3)	454 (47.4)	124 (13.0)	430 (44.9)	152 (15.9)	401 (41.9)	160 (16.7)	334 (34.9)	85 (8.9)
**3**	414 (43.3)	15 (1.6)	369 (38.6)	12 (1.2)	376 (39.3)	13 (1.3)	427 (44.6)	13 (1.4)	290 (30.3)	8 (0.8)
Total	957 (100)	957 (100)	957 (100)	957 (100)	957 (100)	957 (100)	957 (100)	957 (100)	957 (100)	957 (100)
Number reporting presence of problems	844 (88.2)	152 (15.9)	823 (86.0)	136 (14.2)	806 (84.2)	165 (17.2)	828 (86.5)	173 (18.1)	624 (65.2)	93 (9.7)
Change in numbers reporting presence of problems (%)	-692 (-82.0)		-687 (-83.5)		-641 (-79.5)		-655 (-79.1)		-531 (-85.1)	
Rank of dimensions in terms of % changes	3		2		4		5		1	

Level 1 = no problems, Level 2 = some problems, and Level 3 = a lot of problems. Percentages are calculated based on the total number of participants (*N* = 957) at each time point. Negative percentages in the “Change in numbers reporting presence of problems (%)” indicate reductions in reported problems from admission to discharge

The study employed the Paretian Classification of Health Change (PCHC) analysis to assess responsiveness at the individual level across children’s/adolescents’ and their parents’/caregivers’ measures. The findings revealed that a significant majority (*n* = 875, 91.4%) demonstrated health improvement according to PCHC criteria in both measures. Instances of unchanged health status were comparatively rare, accounting for 1.4% in children’s/adolescents’ reports and 2.1% in parents’/caregivers’ reports. The proportion of children/adolescents experiencing a decline in health was remarkably low, documented at 2.9% in the reports of children/adolescents and even lower at 0.4% in the reports of parents/caregivers (Table 7).

Similarly, the evaluation of the responsiveness of child/adolescent and parent/caregiver measures utilizing the EQ-5D-Y-3L index and EQ VAS scores yielded significant findings. The EQ-5D-Y-3L index scores demonstrated substantial increments from admission to follow-up for both measures. Specifically, the mean scores for children/adolescents significantly increased from 0.422 to 0.810 (*p* < 0.01), while those for parents/caregivers increased from 0.350 to 0.844 (*p* < 0.01). Similarly, the EQ VAS scores also exhibited significant changes, increasing from 36.2 to 72.1 for children/adolescents and from 36.3 to 79.5 for parents/caregivers (*p* < 0.01) (Table 8).

**Table 7:** Changes in health for children/adolescents and parents/caregivers reported according to the Paretian classification of health change

Health status	Child/adolescent (%)	Parent/caregiver (%)
No change	13 (1.4)	20 (2.1)
Improve	875 (91.4)	875 (91.4)
Worse	28 (2.9)	4 (0.4)
Mixed change	41 (4.3)	58 (6.1)
Total	957 (100)	957 (100)

**Table 8:** Paired t-test result of difference in mean EQ-5D-Y-3L index and EQ VAS scores in children/adolescents and parents/caregivers

HRQoL		Child/adolescent	t-test *p* value	Parent/caregiver	t-test *p* value
EQ-5D-Y-3L index score	Admission mean (SD)	0.422 (0.286)	< 0.001	0.350 (0.323)	< 0.001
	Follow up mean (SD)	0.810 (0.144)		0.844 (0.112)	
EQ VAS score	Admission mean (SD)	36.2 (18.3)	< 0.001	36.3 (18.2)	< 0.001
	Follow up mean (SD)	72.1 (21.5)		79.5 (18.1)	

HRQoL: Health related quality of life; SD: Standard deviation

## Discussion

This study analyzed data from 957 child/adolescent patient-parent/caregiver dyads. This study is innovative in its approach to examining the convergent validity and responsiveness of the EQ-5D-Y-3L over time through repeated measurements. It aims to make a significant contribution to the understanding of the EQ-5D-Y-3L’s performance in various administration contexts and to provide robust evidence for guiding users of the EQ-5D-Y-3L in diverse settings.

The child/adolescent-reported data on the EQ-5D-Y-3L dimensions indicated that health problems were more frequently reported among older age groups, consistent with existing evidence showing a higher prevalence of reported problems across all dimensions in older children or adolescents [39]. This may be due to age-related increases in emotional insight and self-awareness, which enable older adolescents to recognize and report HRQoL issues more accurately, especially in subjective domains such as pain and emotional well-being. The reported problems were significantly different in many of the EQ-5D dimensions, including LAM, UA, PD, and WSU, with the older age group (12–18 years) consistently reporting the highest frequency of problems. This suggests a need for age-specific interpretation of EQ-5D-Y-3L data, as older adolescents may perceive and express health limitations differently from younger children.

Although gender differences were not statistically significant in most dimensions, both the child/adolescent and parent/caregiver reports indicated a significant association between household income and child/adolescent reported WSU dimension. This may reflect the psychosocial burden associated with financial stressors, which can influence children’s/adolescents’ emotional well-being. Moreover, a statistically significant difference was observed between urban and rural residency with respect to the mobility dimension, as reported by children or adolescents. The insufficiency of family income and rural residence could potentially be correlated with limited access to healthcare and other social services, thereby influencing health status and life satisfaction. In this regard, available evidence suggests that children and adolescents from low-income households encounter greater challenges compared to those from higher-income countries [40].

The agreement between children/adolescents and parents/caregivers regarding reported health status across EQ-5D-Y-3L dimensions at admission and discharge showed moderate overall levels, with a slight improvement from admission to discharge. This change may reflect increased parent/caregiver familiarity with the child’s/adolescent’s condition and observable changes during the hospital stay, which enhance the accuracy of proxy reporting. Parents/caregivers may initially misjudge aspects of the child’s/adolescent’s health due to stress, uncertainty, or limited understanding of symptoms but can become more attuned as treatment progresses. However, agreement was consistently poorer for emotional domains such as the WSU dimension [41]. Since emotional states are inherently subjective and less observable, they are more challenging for parents/caregivers to assess accurately. This discrepancy may be further influenced by caregivers’ anxiety, which can distort their perceptions of their children’s emotional well-being. Studies show that parents/caregivers often misinterpret their children’s feelings, leading to discrepancies between perceived and actual emotional states [42].

Subgroup trends revealed important patterns in agreement. Children/adolescents aged 7–15 years showed the highest agreement levels, likely due to developmental readiness to self-report and stronger parent/caregiver-child/adolescent communication at this stage. In contrast, agreement was lower among the youngest children (4–6 years), possibly due to their limited expressive capacity, and among older adolescents (16–18 years), who may experience greater emotional autonomy and privacy, which limits parental awareness. Boys exhibited a notable decline in agreement at discharge, especially in utility scores, compared to girls. This may reflect gender differences in emotional expression or caregiver expectations. In Ethiopian culture, restrictive gender norms may hinder both boys and girls from expressing vulnerability, as emotional disclosure is often perceived as a sign of weakness [43]. This cultural norm may contribute to underreporting emotional distress and other health concerns, complicating accurate health state reporting [43]. Additionally, urban caregivers showed slightly lower agreement, potentially due to competing demands, reduced time spent with the hospitalized child, or greater reliance on medical staff for updates. These patterns emphasize the importance of considering age, gender, and environmental factors when interpreting agreement between self- and proxy-reports.

The study evaluated the convergent validity of child/adolescent and parent/caregiver-reported EQ-5D-Y-3L index and EQ VAS scores at admission and discharge. Significant correlations (*p* < 0.001) were found, indicating a consistent moderate positive relationship between the EQ-5D-Y-3L index and EQ VAS. This may imply that both EQ-5D-Y-3L and EQ VAS could be used as health outcome measures for healthcare decision-making relevant to the health of children and adolescents. The evidence around this is inadequate in children; however, in the adult population, the convergence has been reported in several studies, and this might be related to some shared construct of these two measures [44].

Child/adolescent and parent/caregiver reports showed increasing correlation coefficients from admission to discharge/follow-up, indicating an improvement in self-perceived health status over time. Specifically, children/adolescents reported a significant improvement in the LAM dimension, with a 76.2% decrease in reported problems, while caregivers highlighted the WSU dimension as showing the most significant improvement, with an 85.1% reduction in reported problems. EQ-5D-Y-3L index scores for both groups showed substantial increases from admission to follow-up, with statistically significant changes (*p* < 0.001). Similarly, EQ VAS scores exhibited significant increases for both children/adolescents and parents/caregivers.

Limitations of this study include, for example, that participants included in this analysis were hospitalized to receive treatment regimens; hence, improvements in HRQoL outcomes were expected as a result of treatment. This might partially explain why the clinical service provided for children worked. Although previous studies have indicated moderate responsiveness of EQ-5D-Y-3L, our study provides additional evidence from a low-income setting to fill a gap in the literature regarding EQ-5D-Y-3L responsiveness in children and caregiver populations in Ethiopia. Furthermore, while all data collectors were trained using a protocol, formal inter-data collector reliability was not assessed. This may introduce a potential source of variation in how responses were administered or recorded. We also acknowledge that cognitive interviews were not conducted to assess the understanding of the EQ-5D-Y-3L items among younger children, and children with developmental disabilities were not included in the sample, which limits the generalizability to the broader pediatric population. Additionally, due to the absence of an Ethiopian EQ-5D-Y-3L value set, the Zimbabwean value set was used for utility estimation. Although this set is regionally more comparable than other available sets, it may introduce some limitations due to cultural and contextual differences. Selection bias is another potential limitation, as patients with more severe illness or lower HRQoL may have been less likely to complete both baseline and discharge assessments, possibly leading to an overestimation of HRQoL improvements. Finally, because HRQoL data were collected during hospitalization, social desirability bias or acute emotional states might have influenced participants’ self- or proxy-reported responses, potentially resulting in under- or over-reporting of problems. Therefore, these limitations should be taken into account when interpreting the findings of this study.

Future studies should focus on how the EQ-5D-Y-3L performs across diverse environments and explore how cultural factors influence proxy-self report agreement, especially norms related to emotional expression and parent/caregiver-child/adolescent communication that can affect health status reports. Additionally, efforts should be directed towards developing locally validated EQ-5D-Y-3L value sets for Ethiopia, using mixed-methods approaches to better understand cultural perceptions of health and quality of life, and conducting longitudinal studies to assess responsiveness over time. Beyond its research implications, these findings underscore the potential for integrating patient-reported outcome measures, such as the EQ-5D-Y-3L, into clinical practice to improve health outcomes. Its simplicity also makes it suitable for adaptation in various settings such as outpatient clinics, rural health initiatives, and school-based health screenings where quick, standardized assessments of child well-being are especially valuable for informing clinical decisions and policy.

## Conclusions

The findings from this study demonstrated moderate agreement between child/adolescent and parent/caregiver assessments and confirmed the tool’s responsiveness to clinical improvements during hospitalization. Older adolescents, those from lower-income households, and rural residents reported more HRQoL problems, underscoring the importance of considering socio-demographic differences in HRQoL assessments. These findings suggest that the EQ-5D-Y-3L can serve as a practical and reliable tool for assessing HRQoL in pediatric populations. Its use in both self- and proxy-reported formats enables more inclusive care, particularly when children are too young or unable to self-report. Integrating the EQ-5D-Y-3L into pediatric care routines and health monitoring systems can help healthcare providers and policymakers identify unmet needs, guide care planning, and track outcomes more consistently, especially in resource-limited settings. Future studies should validate the EQ-5D-Y-3L in other settings and among different pediatric populations, and explore how cultural norms, parent/caregiver-child/adolescent communication, and health literacy affect reporting and interpretation of proxy-reported outcomes.

## Data Availability

The data analyzed for the current study is available from the corresponding author upon reasonable request.

## Abbreviations

EQ-5D-Y-3L: EQ-5D Youth Version with 3 Levels
EQ VAS: EuroQol Visual Analogue Scale
HRQoL: Health-Related Quality of Life
ICC: Intraclass Correlation Coefficient
MO: Mobility
LAM: Looking After Myself
UA: Doing Usual Activities
PD: Pain/Discomfort
WSU: Feeling Worried, Sad or Unhappy
